# Predicting chronic pain using wearable devices: a scoping review of sensor capabilities, data security, and standards compliance

**DOI:** 10.3389/fdgth.2025.1581285

**Published:** 2025-05-22

**Authors:** Johannes C. Ayena, Amina Bouayed, Myriam Ben Arous, Youssef Ouakrim, Karim Loulou, Darine Ameyed, Isabelle Savard, Leila El Kamel, Neila Mezghani

**Affiliations:** ^1^ Applied Artificial Intelligence Institute (I2A), TELUQ University, Montreal, QC, Canada; ^2^ Open Innovation Laboratory in Health Technologies, CHUM Research Center, Montreal, QC, Canada; ^3^ Department of Computer Science and Mathematics, University of Quebec at Chicoutimi, Chicoutimi, QC, Canada

**Keywords:** chronic pain, wearable device, privacy, standardization, predictive analytics

## Abstract

**Background:**

Wearable devices offer innovative solutions for chronic pain (CP) management by enabling real-time monitoring and personalized pain control. Although they are increasingly used to monitor pain-related parameters, their potential for predicting CP progression remains underutilized. Current studies focus mainly on correlations between data and pain levels, but rarely use this information for accurate prediction.

**Objective:**

This study aims to review recent advancements in wearable technology for CP management, emphasizing the integration of multimodal data, sensor quality, compliance with data security standards, and the effectiveness of predictive models in identifying CP episodes.

**Methods:**

A systematic search across six major databases identified studies evaluating wearable devices designed to collect pain-related parameters and predict CP. Data extraction focused on device types, sensor quality, compliance with health standards, and the predictive algorithms employed.

**Results:**

Wearable devices show promise in correlating physiological markers with CP, but few studies integrate predictive models. Random Forest and multilevel models have demonstrated consistent performance, while advanced models like Convolutional Neural Network-Long Short-Term Memory have faced challenges with data quality and computational demands. Despite compliance with regulations like General Data Protection Regulation and ISO standards, data security and privacy concerns persist. Additionally, the integration of multimodal data, including physiological, psychological, and demographic factors, remains underexplored, presenting an opportunity to improve prediction accuracy.

**Conclusions:**

Future research should prioritize developing robust predictive models, standardizing data protocols, and addressing security and privacy concerns to maximize wearable devices’ potential in CP management. Enhancing real-time capabilities and fostering interdisciplinary collaborations will improve clinical applicability, enabling personalized and preventive pain management.

## 1 Introduction

Chronic Pain (CP) is a major public health issue affecting millions of people worldwide, causing significant physical, emotional, and financial challenges (1, 2). Approximately 10% of the global population experiences CP (3, 4), with some studies reporting prevalence rates between 20% and 77% in certain countries (5–9). CP is often associated with various health problems, including musculoskeletal disorders, neuropathies, and chronic diseases, which frequently reduce quality of life and impair functionality. According to the World Health Organization, the most common form of CP is low back pain, which has impacted 619 million people in 2020. This number will increase to 843 million by 2050, primarily due to population growth and aging (10). Traditional methods for reducing CP, such as medications, interventional procedures, physical and psychological therapies, and lifestyle approaches have shown limited long-term effectiveness (2, 11). These approaches lack real-time monitoring, may cause side effects, and are not always tailored to individual pain patterns. This reinforces the need for more effective and customized solutions.

Wearable technologies are emerging as innovative, non-intrusive solutions to pain management. They enable real-time data collection, continuous monitoring, enhanced patient engagement, and reduced dependence on pharmaceuticals (12, 13). Such technologies have been shown to improve communication between patients and healthcare providers (14). However, despite the increasing number of research on digital tools for managing CP, existing studies have primarily focused on detecting pain severity (15–17) and improving adherence to prescribed treatments (18–20). Previous article reviews provided an overview of digital technologies for pain management. Although they reported various physiological signals such as heart rate, muscle activity, and sleep patterns (16, 21, 22) for pain monitoring, and explored the usability and feasibility of health tools (23–25), they do not adequately address the predictive capabilities of these technologies. Many of them emphasize the correlation between pain and physiological data without addressing the quality of the data collected by the different types of sensors and the predictive potential of the wearable device, in order to anticipate CP episodes. Furthermore, most studies are limited to controlled settings, bypassing the importance of real-world data and longitudinal analysis for proactive CP management.

This review focuses on predictive models for CP using wearable devices by highlighting how these technologies can not only monitor CP but also anticipate its intensity through multimodal data. Indeed, pain is a complex phenomenon, encompassing sensory perception as well as behavioral, physiological and psychological responses (26). Therefore, its management requires a multidisciplinary approach, involving expertise in medicine, psychology, physiotherapy and social sciences for a comprehensive understanding and treatment. Another key aspect of this study's contribution is the priority given to data security, privacy protection, and compliance with health data standards in the use of these devices. While much research has explored the usability and technical aspects of wearable devices (21), there is an insufficient consideration given to the security and privacy of sensitive health information. With the rise of cyberattacks and the increasing need to safeguard personal data, data security and privacy in healthcare systems have become crucial (27, 28). This presents a major challenge, as both device and software security must be ensured. Addressing these factors is essential for developing effective tools for CP management. Sensor capabilities, data security, and standards compliance are closely interconnected in the context of predicting CP using wearable devices. Synergy between these aspects is essential to ensure the accuracy of the collected data, its protection against unauthorized access, and its use in compliance with regulatory requirements.

This scoping review presents a novel framework for managing CP by examining device technologies, addressing legal considerations such as data privacy, regulatory compliance, and ethical concerns, and highlighting predictive modeling based on multimodal data from wearable sensors. To the best of our knowledge, no review published to date has emphasized the importance of combining various types of data (physiological, behavioral, environmental factors, etc.) to improve the accuracy and reliability of CP prediction models while deepening the data security and privacy requirements. In doing so, we aim to provide more robust methods for secure, compliant, and comprehensive pain management tools, ultimately benefiting designers, users and healthcare providers.

## 2 Methods

To systematically analyze studies focusing on CP and wearable devices, this review was conducted in accordance with the Preferred Reporting Items for Systematic Reviews and Meta-Analysis-Scoping Review (PRISMA-ScR) guidelines (29). We followed the structure defined by Arksey and O'Malley (30), comprising five stages: (1) defining the research question; (2) identifying relevant studies; (3) establishing eligibility criteria for study selection; (4) charting the data; (5) summarizing and reporting the results. Adhering to these stages facilitated a comprehensive mapping of the literature and a synthesis of findings related to CP prediction.

### 2.1 Review questions

This review aims to address the following questions regarding technologies designed to collect data related to CP: (a) Which wearable devices, through their algorithms and monitoring capabilities, can help detect or prevent chronic pain? (b) How do these devices comply with regulatory standards to ensure data security and protect user privacy? (c) What key features contribute to their effectiveness for chronic pain prediction?

### 2.2 Identifying relevant studies

The search strategy was developed using keywords and terms existing on pain management technologies. It included three main steps: step 1) identifying terms related to chronic pain (e.g., persistent pain); step 2) defining terms related to wearable devices (e.g., wearable technology); and step 3) combining parameters from steps 1 and 2 to retrieve references covering both concepts. Studies were extracted across six databases (PubMed, Web of Science, Scopus, Engineering Village, IEEE Xplore, and Google Scholar) from the inception of the database to November 4, 2024, with no limit on publication year applied. The first 155 results from Google Scholar and all identified studies from the other databases were imported into Covidence systematic review software (31) for duplicate removal. Furthermore, an additional manual search of reference lists within relevant systematic reviews was conducted. The search strategy was tailored to each database, giving priority to articles in English or French. The detailed search strategy is presented in Supplementary Material.

### 2.3 Eligibility criteria

Eligible studies for addressing our third review question must include participants experiencing chronic pain, defined by the International Association for the Study of Pain (IASP), as “pain that persists or recurs for longer than three months” (32). Only studies utilizing wearable devices for CP prediction were included. Studies involving invasive technologies, non-autonomous systems, robotic systems or exoskeletons, and studies only exploring relationships between biometrics data (e.g., heart rate variability, skin conductance) and CP without developing predictive models were excluded. To extend the literature scope and guide future research, all experimental study designs were considered. However, protocols, reviews, books, abstracts, editorials, commentaries, dissertations, and poster presentations were excluded. In cases where the same author has multiple publications on this review topic, the publication with the most comprehensive and updated data was prioritized while ensuring relevance to the scoping review objectives. Studies were also excluded if they focused on activity recognition or virtual reality systems, or had other purposes.

### 2.4 Data charting

The relevant information was extracted and synthesized: (1) authors, publication year and country; (2) sample size and participants characteristics; (3) intervention settings (e.g., hospital, home, or laboratory); (4) type of the wearable device used; (5) prediction models and their accuracy; (6) outcomes, focusing on technical effectiveness, usability, data security, and conformity to relevant norms and standards.

### 2.5 Summary and report of the results

The summary and report included an overview of current wearable devices described in the literature, the representation of the most used prediction models, sensing elements and biometric variables across the extracted studies. Additionally, a table is included to illustrate how these devices align with different health standards. An in-depth analysis of the included studies was conducted to identify limitations in the predictive models, thereby providing valuable information to guide future research.

## 3 Results

**Figure 1:**
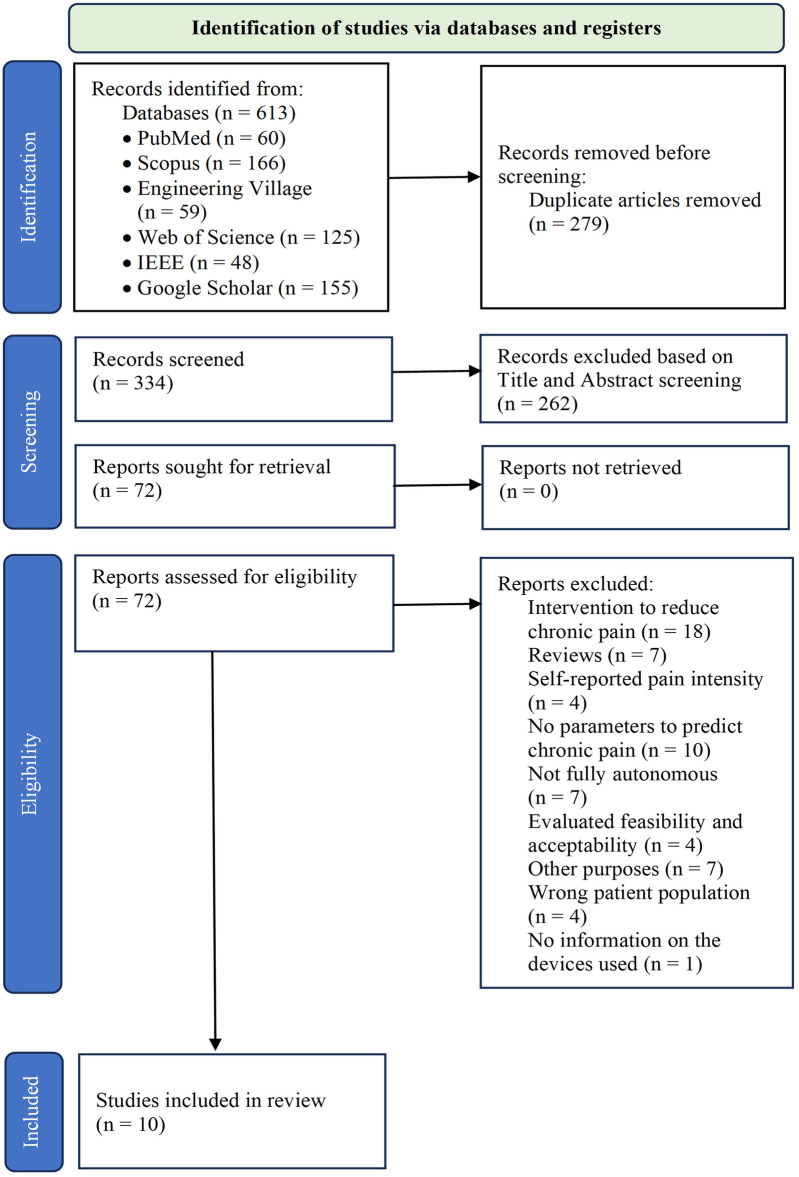
PRISMA flow chart for study selection.

The literature search identified 613 references, which were screened for relevance. After removing duplicates, 334 studies were retained for further analysis. Of these, 72 studies were eligible for full-text review. Sixty-two (62) studies were excluded from the analysis for several reasons: they either did not use recorded parameters to predict pain, did not involve patients with CP, or focused exclusively on interventions aimed at reducing CP (e.g., physical therapy, medication trials) without addressing predictive methodologies, such as the use of predictive algorithms. As a result, 10 studies met the inclusion criteria for this scoping review. The study selection process is detailed in the flowchart presented in Figure 1.

### 3.1 Sensing elements and predictive models for chronic pain

**Figure 2:**
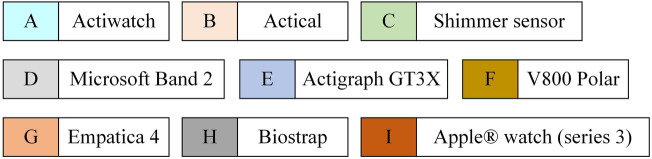
Overview of wearable chronic pain management devices. For more information on these devices, please refer to the following ref. [21, 38, 40, 42, 43, 45, 64].

**Figure 3:**
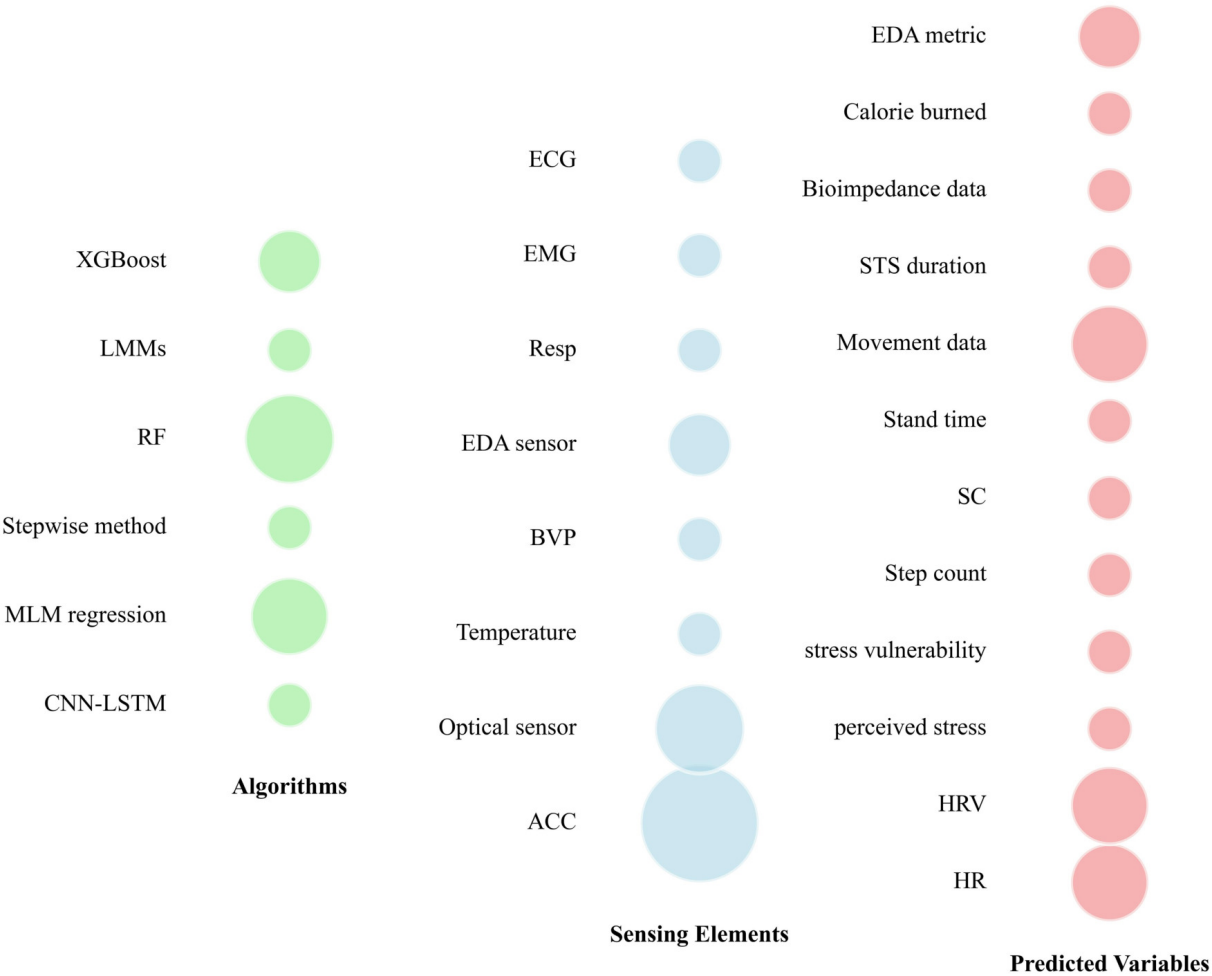
Predictive models, sensors, and variables used across literature for predicting chronic pain. CNN-LSTM, convolutional neural network-long short-term memory; MLM, multilevel model; RF, random forest; LMMs, linear mixed-effects models; ACC, accelerometer; BVP, blood volume pulse; EDA, electrodermal activity; Resp, respiration sensor; EMG, electromyography; ECG, electrocardiogram; HR, heart rate; HRV, heart rate variability; SC, skin conductance; STS: sit-to-stand.

Different tools have been used over the past three years in scientific papers for CP prediction/detection (Figure 2). Wearable sensors, including accelerometers and optical sensors, enable real-time monitoring of physiological parameters such as heart rate and step count. These advancements not only aid in the prediction of CP but also enhance patient engagement and preventive health management. Figure 3 illustrates the various algorithms, sensing elements, and predicted variables used in CP prediction. Methods such as Random Forest algorithms, accelerometer sensors, and key variables like movement intensity, heart rate, heart rate variability, and electrodermal activity (EDA) have shown particular effectiveness. These approaches provide valuable information into CP intensity and its modulation, which could enable more precise and personalized pain management strategies.

### 3.2 Multimodal for chronic pain prediction

**Figure 4:**
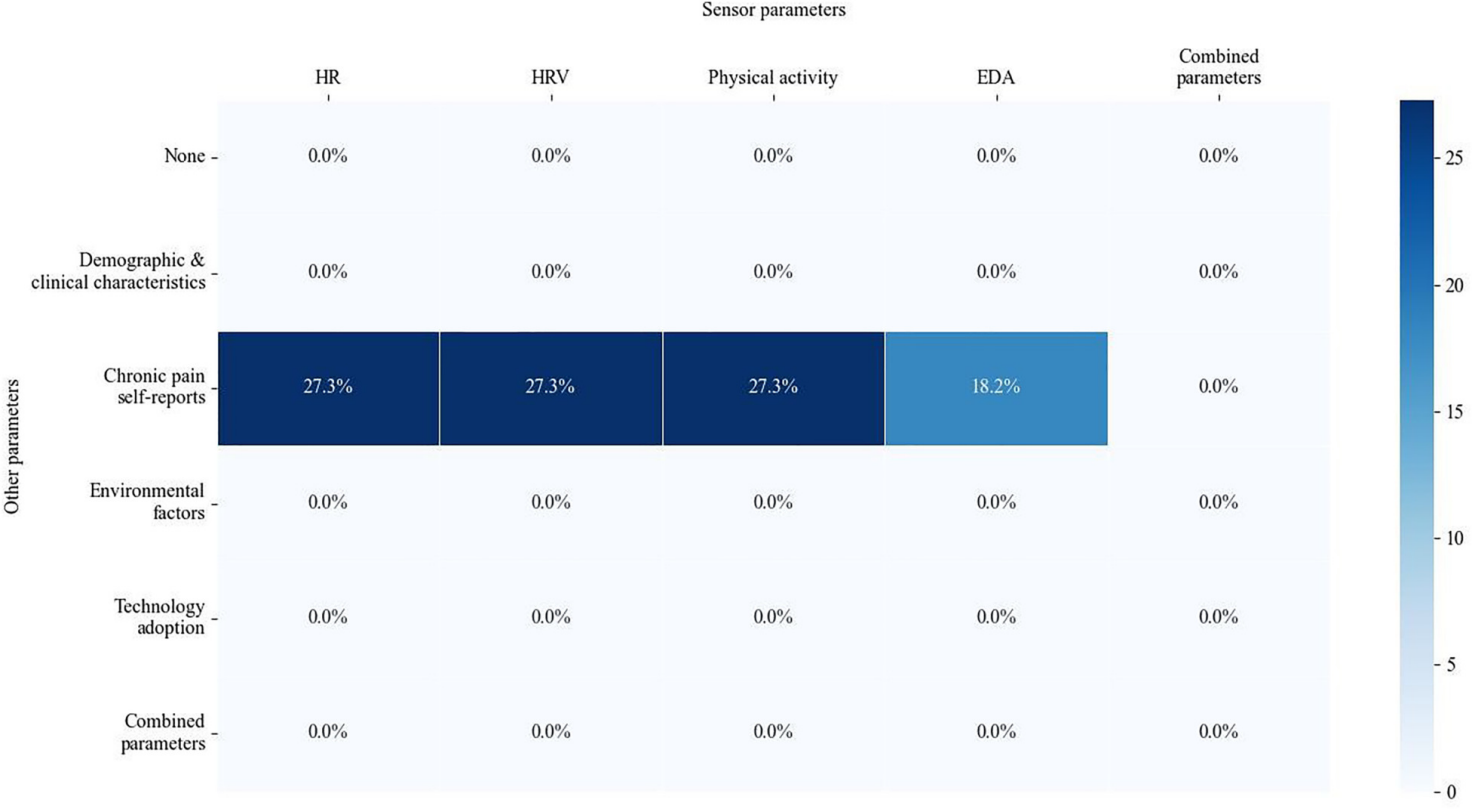
Percentage of studies by parameter combination for chronic assessment purpose. HR, heart rate; HRV, heart rate variability; EDA, electrodermal activity.

The studies summarized in this scoping review have achieved varying levels of accuracy, using a number of technologies (Figure 2) and models (Table 1). More than half of the studies focused on patients with an average age above 40 years (Table 1). Notably, one study was conducted in a naturalistic setting, collecting data from 688 patients with CP. The remaining were conducted in clinical, home and/or laboratory settings. A variety of subjective pain scales, including self-report measures such as the Visual Analog Scale (VAS), the McGill Pain Questionnaire, and the Brief Pain Inventory, are employed by these studies to validate pain intensity. In addition, our scoping review specifically examined how physiological data were combined to other parameters for predicting pain intensity. While some studies demonstrate a combination of specific physiological markers correlated with self-reports pain, no study has revealed how factors such as demographic and clinical characteristics, environmental factors, and technology adoption can provide more comprehensive information on chronic pain prediction (Figure 4). Table 2 summarizes the use of multimodal inputs and highlights their potential complementary contributions to CP prediction, emphasizing the missed opportunity for more integrated modeling approaches in the current literature.

**Table 1:** Study characteristics and model performance for chronic pain prediction.

Author (ref)	Year	Country	Participants’ characteristics & Settings			CP prediction features			Outcomes
			Sample size (health status)	Age in years (mean ± SD or Range)	Settings	Wearable Type	Model performance	Correlation scale	
Dudareva et al. (40)	2024	Canada	66 (with CP)	22–79	Home	Wrist-worn Biostrap	(t-test) b = 0.012, *P* = 0.005	Standard 11-point pain scale	A predictive relationship between sleep heart rate and next-day pain intensity (*P* < 0.05).
Dorris et al. (46)	2024	USA	688 (with CP) and 3552 (without CP)	49.54 ± 18.38	Naturalistic setting	ActiGraph (AM-7164)	AUC validation = 0.60, AUC test = 0.57	Pain Questionnaire (home interviews)	CP could be predicted using daily activity movement intensity.
Gungormus et al. (38)	2024	Spain	67 (adults with rheumatic disease)	65 — 90	Multicenter	Empatica E4	*p* = 0.001, adjusted *R^2^* = 0.154	McGill Pain Questionnaire	Significant predictive values of HRV, SC, perceived stress, and stress vulnerability in relation to pain qualities and thresholds.
Patterson et al. (39)	2023	USA	15 (twelve years of CP)	52.25 ± 9.7	Home and Clinical	Apple ® Watch (Series 3)	Accuracy = 0.768 ± 0.012	Numerical rating scale	The model could predict the pain intensity of mild, moderate, and severe.
Perraudin et al. (62)	2018	Ireland	30 (with arthritis) 15 (healthy participants)	31 — 75	Home	ActiGraph GT9X	N/A	Patient report outcomes containing 0–10 numerical scale	Able to predict the severity of morning pain and stiffness via 5×STS duration, disease type, and gender.
Critcher et al. (63)	2023	USA	20 (with and without knee osteoarthritis**)**	73.5 ± 8.26	N/A	Smart knee brace	*P* < 0.05	Monthly pain self-reported	Increased resistance and decreased reactance per unit length were linked to higher knee pain risk.
Luebke et al. (41)	2023	Germany	72 (52 with CP and 20 healthy subjects)	18 — 65	Laboratory	Empatica E4, respiBAN, and Electrodes	91.67% (using 31 features)	Visual Analogue Scale	Electrodermal activity is the best marker for distinguishing between low and high pain levels.
Jacobson et al. (48)	2021	USA	68 (54.41% with CP)	41.28 ± 8.11	Clinical	Actigraph	74.63% accuracy	Brief Pain Inventory	Results suggest that digital biomarkers can predict pain severity, pain chronicity, and worry severity with high precision.
Sett et al. (45)	2019	Switherland	45 (30 arthritis patients and 15 healthy volunteers)	47.5 ± 4.5	Home	Actigraph GT3X Link	AUC: 0.79 (*±*0.18)	Pain was recorded on a 0–10 scale	Physical activity is correlated with pain and stiffness and can predict daily pain of arthritis patients.
Stojancic et al. (64)	2023	USA	20 (with sickle cell disease)	30 — 41	Clinical	Apple® Watch (series 3)	37.72% 69.06% 84.52%	11-point pain scale	The best-performing model was the random forest model, which was able to predict the pain scores with an accuracy of 84.5%, and a RMSE of 0.84.

AUC, area under the curve; N/A, not available; STS, sit-to-stand; RMSE, root mean square error; CP, chronic pain; HRV, heart rate variability; SC, skin conductance.

**Table 2:** Summary of multimodal data sources and their potential contributions to CP prediction.

Modality	Sensors/sources	Features	Potential contribution to CP prediction
Physiological	Wearables, biosensors (e.g., EDA sensors)	HR, HRV, SC, activity level, sleep metrics	Captures real-time physical responses to CP
Demographic	Surveys, EHR	Age, sex, ethnicity, education level, employment	Provides baseline risk factors and stratification across populations
Clinical	Medical records, clinician input	Pain history, comorbidities, medications, diagnoses	Offers diagnostic context and longitudinal health status
Psychological	Questionnaires (e.g., BDI)	Anxiety, depression, catastrophizing, mood	Adds subjective and cognitive dimensions of pain experience
Behavioral	Smartphone use, app logs, passive monitoring	Sleep patterns, mobility, communication, app interactions	Reflects daily functioning, lifestyle, and behavioral adaptations
Environmental/Contextual	GPS, weather data, social context logs	Location, ambient temperature, social setting	Accounts for external triggers and modulators of CP
Self-reported	Pain surveys	Pain intensity, duration, interference, triggers	Provides subjective ground truth and context-rich annotations

CP, Chronic Pain; HR, heart rate; HRV, heart rate variability; EHR, Electronic Health Record; BDI, Beck Depression Inventory; GPS, Global Positioning System; EDA, Electrodermal Activity; SC, skin conductance.

### 3.3 Data security, privacy and standards compliance in wearable devices for CP

While all these devices (Figure 2) are wearable smart devices, they collect, store, process, treat, and deliver data. These data are considered personal data and must be protected and handled securely to ensure the privacy of healthcare information. For these reasons, many industry standards and regulations, presented in Table 3, provide guidelines and techniques to ensure data security in connected devices, including healthcare devices. Indeed, industrial standards like ISO 27001 require conformity with techniques such as data encryption, user authentication, data masking, access control, secure development lifecycle, backup and restore management, protection against malware, equipment maintenance, and compliance with data protection regulations. Table 4 outlines the challenges, methods as well as some recommendations that should be considered when using those techniques. These recommendations are based on several different sources, showing the different challenges and methods of the techniques used. Personal data protection regulations such as the GDPR (General Data Protection Regulation) require that individuals whose data is being collected must be informed about the data being collected, and they must be able to access, rectify, or delete it. The regulation also defines restrictions and special requirements for data transferred both locally and internationally. GDPR mandates that data collection be minimized to what is strictly necessary for the intended purposes. The results reported in Table 5 show the conformity of each device with one or more of the mentioned industrial standards and regulations.

**Table 3:** List of industry standards and regulations.

Standard / Regulation	Description
ISO13485	Medical devices — Quality management systems — Requirements for regulatory purposes
ISO27001	Information security, cybersecurity and privacy protection — Information security management systems — Requirements
ISO27701	Security techniques — Extension to ISO/IEC 27001 and ISO/IEC 27002 for privacy information management — Requirements and guidelines
ISO62304	Medical device software — Software life cycle processes
GDPR	General Data Protection Regulation (Europe)
HIPAA	Health Insurance Portability and Accountability Act (United States)
PIPEDA	The Personal Information Protection and Electronic Documents Act (Canada)
NIST SP 800-53	Security and Privacy Controls for Information Systems and Organizations
FIPS 140-3	a U.S. government computer security standard used to approve cryptographic modules

**Table 4:** Challenges and recommendations for wearable devices used in chronic pain prediction.

Theme	Challenges	Methods	Recommendations
Data encryption	Encryption is not useful when: – Using weak keys – Key management are uncontrolled – Used algorithms are vulnerable	– Symmetric Encryption Method: Using the same private key to encrypt and decrypt data. – Asymmetric Encryption Method: Using both public key and private key to encrypt and decrypt data. Used keys are mathematically linked. – Hybrid Encryption Method: is a combination between symmetric and asymmetric encryption method (65).	For healthcare connected systems, it is recommended to use Hybrid Encryption Method (66).
User authentication	Each method of user authentication has its own disadvantages. But, in general all authentication systems can sometimes fail, locking out legitimate users or, conversely, failing to block unauthorized access due to technical glitches.	– Password based authentication – Multifactor authentication – Biometric authentication – Single Sign-on authentication – Token based authentication – Certificate based authentication (67)	For connected devices included healthcare devices, it is recommended to use: – Password based authentication – Multifactor authentication – Certificate based authentication (68)
Data masking	Data masking is not useful when: – Data loss due to inaccurate data masking – Incomplete data protection due to the potential for reverse engineering of datasets	– Data Pseudonymization – Data Substitution – Data Scrambling – Data Shuffling (69, 70)	N/A
Access control	– Excessive permissions and exceptions – Choosing the appropriate access control model to guarantee adequate security and employee productivity.	– The Mandatory Access Control (71) – The Role-Based Access Control – The Discretionary Access Control – The fourth and final access control model is Rule-Based Access Control	N/A
Data minimization	The most popular challenge with data minimization is to know what information you need to collect.	N/A	N/A
Secure Development Life Cycle	Taken into consideration security aspects in all the development life cycle steps.	No specific method, we must take into consideration security aspect in the development life cycle from requirement to deployment.	N/A
Backups and restore management	Backups and restore may require a lot of time when it is done manually and may need a lot of storage when you have a big amount of data.	Backup management (72) – Cloud Storage – 3-2-1 Backup rule – Hybrid backup solutions – Periodic automated backup – Onsite backup	N/A
Protection against malware	The major downside to any antivirus software is that it can't protect your devices and data from every attack, as various types of threats are constantly evolving and being created.	– Install anti-malware software on the devices (73) – Apply threat detection and response procedures to identify malware and prevent it from spreading – Ensure that files uploaded are properly scanned – Implement security at the web browser level	Install anti-malware software on the devices
Software/hardware maintenance	– Cost: Software maintenance can be time-consuming and expensive and may require significant resources and expertise. – Schedule disruptions: Maintenance can cause disruptions to the normal schedule and operations of the software, leading to potential downtime and inconvenience.	– Corrective Maintenance (74) – Adaptive Maintenance – Perfective Maintenance – Preventive Maintenance	– Perfective Maintenance – Preventive Maintenance (75)

N/A, not available.

**Table 5:** Health standards applied to the wearable devices displayed in Figure 2.

Device	HIPAA	GDPR	ISO 27001	ISO 27701	NIST SP 800-53	FIPS 140-3	ISO 13485	ISO 62304
Actiwatch 2	✘	✔	✔	✔	✔	✘	✘	✔
Actical	✘	✔	✔	✔	✔	✘	✘	✔
Shimmer sensor	✔	✔	✘	✘	✘	✘	✔	✘
Polar V800	✘	✔	✘	✘	✘	✘	✘	✘
Actigraph GT3X	✘	✔	✔	✔	✘	✘	✔	✘
Empatica 4	✔	✔	✔	✘	✘	✘	✔	✘
Apple Watch S3	✘	✔	✔	✘	✘	✔	✘	✘

HIPAA, health insurance portability and accountability Act; GDPR, general data protection regulation; FIPS, federal information processing standards; NIST, national institute of standards and technology.

## 4 Discussion

Advancements in artificial intelligence and predictive analytics offer promising opportunities for predicting chronic pain (CP) intensity and tailoring interventions. In the following subsections, we discuss various aspects involved in designing wearable devices for managing CP.

### 4.1 Sensing elements

Sensing elements play a crucial role in acquiring biometric and environmental data. Our findings reveal that certain sensors, such as the accelerometer and optical sensors, are widely used (Figure 3), underlining their importance in pain management. These sensors seem to be preferred for their availability, ease of integration into wearable devices and ability to provide reliable data in real time. For example, accelerometer are essential for applications such as step counting and posture analysis (33–36), while optical sensors provide precise heart rate measurements using photoplethysmographic methods (37–40). In contrast, other sensors such as temperature, ECG, and EMG, although valuable in specific contexts, are less commonly used (Figure 3). This may be due to constraints such as higher cost, larger size, or probably the complexity of interpreting the data they generate. Finally, the use of specialized sensors, such as EDA sensors, reflects a growing interest in psychophysiological measures such as perceived stress (38, 41). The use of such type of sensor (EDA) indicates a trend towards more focused sensors for CP assessment. Overall, our findings underline the evolution of demand for advanced instrumentation, with an emphasis on the preference for versatile and easily integrable sensors. This present scoping review also suggests a reconsideration of design strategies to incorporate specialized sensors where they can enhance predictive models.

### 4.2 Sensor data quality

When assessing the quality of sensor data for CP monitoring, only devices such as Empatica 4, Polar V800, and Shimmer Sensor (Figure 2) appear to provide features that are suited to tracking physiological and behavioral parameters associated with pain (42, 43). Indeed, high-quality sensors ensure accurate computation of critical parameters such as heart rate variability, skin conductance, movement patterns, and sleep quality, which are critical indicators in chronic pain studies (38, 41). Moreover, Shimmer sensor and Empatica 4, which comply with standards such as ISO 13485 and HIPAA (Health Insurance Portability and Accountability), demonstrate the reliability and accuracy required for medical-grade applications (Table 5). Specifically, Empatica 4 stands out in particular in the field of continuous monitoring, thanks to its ability to detect substantial changes in stress and autonomic responses, facilitating the assessment of CP (38, 41). The accurate and consistent data provided by these sensors offer valuable information into pain triggers, individual response patterns, and treatment effectiveness, making them indispensable tools for researchers and clinicians in CP management.

### 4.3 Predictive models for chronic pain

Random Forest (RF) demonstrated strong performance across multiple studies, achieving notable accuracy levels such as 0.768 ± 0.012 (39); 91.67% (41) and 84.52% (44) (Figure 3, Table 1). This consistency highlights its efficiency in handling large datasets with non-linear relationships. In addition, its area under the curve (AUC) of 0.79 ± 0.18 in one study (45) demonstrates its robustness in classifying pain vs. no pain, particularly in scenarios requiring a balance between sensitivity and specificity. Multilevel Model (MLM) regression also shows significant predictive power, with a t-test coefficient of 0.012 and *p*-value of 0.005, indicating its statistical reliability in capturing relevant data patterns for CP prediction (40). Similarly, the stepwise method, although less used across studies (Figure 3), achieved an adjusted R² of 0.154, *p* = 0.001 (Table 1), indicating moderate predictive ability in the dataset used (38). In contrast, deep learning methods such as Convolutional Neural Network-Long Short-Term Memory (CNN-LSTM) produced weaker results, with AUC values of 0.60 for validation and 0.57 for testing (46), which may reflect challenges in optimizing these models. However, their ability to extract features and model time series suggests that, with improved data quality or tuning, they could provide more significant advantages (47). XGBoost delivered an accuracy of 74.63%, demonstrating its reliability in scenarios with well-preprocessed datasets (48). This performance makes it a viable alternative for real-time CP prediction. Our review highlights the trade-offs inherent in the choice of predictive algorithms. Indeed, for predicting CP, we can note that traditional machine learning models such as RF and MLM regression are more dominant (Figure 3), likely due to their reliability and ease of implementation. However, advanced techniques such as CNN-LSTM and XGBoost are also becoming more popular, especially in scenarios requiring computational efficiency (49). Our results underline the importance of aligning algorithm selection with the specific characteristics of the dataset and the prediction of CP.

### 4.4 Multimodal data integration

This study also emphasizes the crucial importance of integrating multimodal data to improve the prediction of CP. While physiological data such as HR, HRV, physical activity and EDA present objective information (41, 45), they may fail to capture the subjective and contextual dimensions of pain. Similarly, demographic and clinical characteristics present essential information, but lack specificity when used alone. The absence of studies (Figure 4) exploiting fully combined parameters shows a missed opportunity to model the complex and multifaceted nature of CP. Combining diverse data sources could improve predictive accuracy by capturing the interplay between biological, psychological and environmental factors, ultimately leading to more personalized and effective CP management strategies.

To facilitate this integration, a structured approach is necessary. Multimodal data can be processed using advanced modeling techniques such as feature-level fusion (e.g., concatenating features before model input) (50), decision-level fusion (e.g., ensembling predictions from unimodal models) (51), or representation learning approaches like deep learning, which can automatically capture cross-modal interactions (52). For example, synchronizing physiological signals with self-report pain ratings (Table 1) could provide a more comprehensive view of CP experience. Table 2 summarizes the various data types and their potential contributions to CP prediction, highlighting how each modality could add unique value. Such integration will not only enhance model interpretability and generalizability, but also enable the way for real-time, adaptive systems capable of supporting dynamic, personalized CP management.

### 4.5 Data security, privacy and compliance with health standards

Manufacturers like Philips, Shimmer, Polar, ActiGraph, Empatica and Apple, reported their compliance with several industry standards and regulations related to data security and privacy. However, companies like Microsoft and Biosignalsplux, have not reported any compliance with any of the mentioned standards and regulations (Table 3). Table 5 summarizes the compliance status of different devices with the industry standards and regulations.

For Actiwatch2 and Actical, Philips, the manufacturer, mentioned several implemented measures to protect personal data and ensure all data security collected by those devices (53). For shimmer sensors and Polar V800, the manufacturer does not have access to the data. All collected, transferred and stocked data are handled by the end user. When a device is returned for maintenance, the manufacturer ensures that all data is immediately deleted. For these reasons, the applicable techniques and methods (Table 4) are not relevant. Thus, there is no information about the maintenance of the software and the hardware (54). The Microsoft band 2 lacks detailed documentation since it is no longer available on the market. We have not found any reliable information about the techniques and methods used. ActiGraph GTX3 and Empatica4, both of the manufacturers, ensure that they take reasonable and appropriate measures to protect personal data, but there is not enough information for the used methods (55, 56). Regarding the Apple Watch Series 3, Apple decided that this model will no longer receive updates to watchOs 9, and support for watchOs 8 will also be discontinued (57). So, this device becomes vulnerable.

Two main issues arise across these devices. First, there is a huge amount of data to be stored and transferred which increases the complexity of the data security process. Second, devices like Biosignalsplux, which do not provide data storage in cloud or in-premise servers, place the recorded data in the hands of the end user. Despite compliance with industrial standards and regulations by manufacturers, data privacy and protection continue to be at risk, as end users, including researchers, must also follow the good practices described in Table 4.

### 4.6 Challenges and perspectives for improvement

Beyond the promise of technology integration in CP management, significant challenges remain in realizing the full potential of these innovations. Data privacy concerns, interoperability issues between different systems and disparities in access to technology are key obstacles to the widespread adoption of technological CP management solutions. The need to ensure that patient data is protected and that technologies work smoothly across different platforms is essential to building trust and efficiency (58, 59). Similarly, in the context of predictive algorithms and sensing elements, there are notable challenges. One of the main issues is the variability in model performance across studies, as shown by the large fluctuations in the accuracy of models, whose results range from 37.72% to 91.67% (Table 1). Furthermore, the CNN-LSTM model showed low AUC values ​​(0.60 for validation and 0.57 for testing), highlighting the need for greater consistency in predictive capabilities. These inconsistencies underline the need to improve the quality and standardization of the datasets used for training and validation to ensure more reliable and reproducible results. Furthermore, the computational complexity of advanced models such as CNN-LSTM could limit their applicability in real-time systems, particularly in resource-constrained environments. Additionally, integrating various sensing elements such as EDA, ECG, accelerometers, and others presents challenges related to sensor fusion and data synchronization (60). These issues can affect prediction accuracy, making it difficult to achieve consistent and meaningful results across diverse populations and conditions.

To overcome these challenges, future research should focus on a variety of strategies. Standardizing data collection and preprocessing will help ensure data quality and comparability, facilitating the training of more robust and generalizable models. Optimizing feature selection techniques, tailored specifically to the characteristics of the collected data, is essential to improve the performance of predictive algorithms (41). Adapting models to the specific application will help improve their relevance and effectiveness in the real world (52). Refining the real-time capabilities of these systems is also crucial, as the ability to provide immediate feedback based on continuous data streams could greatly improve patient outcomes. Moreover, promoting interdisciplinary collaboration between data scientists, clinicians, engineers, and healthcare providers will be crucial to develop more practical and reliable predictive systems that can be integrated into clinical practice.

Longitudinal studies are needed to investigate the temporal relationships between physiological changes and CP episodes. Such research would help to understand how individual physiological profiles change over time and how these changes correlate with CP outcomes (61). Personalized algorithms that predict CP outcomes based on these profiles could enable great potential to improve pain management by tailoring interventions to the unique needs of each patient. In addition, emerging technologies, such as wearable sensors and advanced machine learning algorithms (21), provide exciting opportunities for real-time monitoring. These innovations could enable continuous monitoring of pain-related physiological parameters, allowing for more dynamic and responsive pain management strategies that adapt to the needs of the individual in real time. However, integrating these technologies into clinical practice will require overcoming existing barriers and ensuring that the systems are both clinically effective and accessible to diverse populations. Moreover, compliance with industry standards and regulations (Table 3) will be essential to ensure data security and privacy.

## Conclusion

Wearable devices offer significant potential for chronic pain (CP) management through real-time monitoring and personalized treatments. However, their ability to predict the evolution of CP remains limited, as most studies focus on the correlation between physiological markers and CP rather than predicting CP episodes. In this scoping review, we focused on predictive models for CP as well as the possibility of integrating multimodal data, combining physiological, psychological, and demographic factors. While models such as Random Forest are promising, more complex algorithms face challenges related to data quality and computational limitations. Data security and privacy also remain major concerns. Although many proposed devices for CP adhere to regulations such as GDPR and ISO, there are still gaps in user data protection. Future research should focus on developing robust predictive models, addressing security issues, and standardizing data protocols. These efforts will enhance the efficacy and clinical applicability of wearable devices, facilitating more efficient and personalized pain management solutions.

## Data Availability

The original contributions presented in the study are included in the article/Supplementary Material, further inquiries can be directed to the corresponding author.
