# Supplementary material for: Predicting chronic pain using wearable devices: a scoping review of sensor capabilities, data security, and standards compliance
Source: Front Digit Health. 2025 May 22;7:1581285. doi: 10.3389/fdgth.2025.1581285 (PMC12137249; doi:10.3389/fdgth.2025.1581285)
Supplement: Supplementary file 1 [file Table1.docx]

Supplementary Material

# Supplementary Table

**Table: Detailed search strategy**

**PubMed**

| **3** | #1 AND #2 |
| --- | --- |
| **2** | ("Wearable Electronic Devices"[mh] OR wearable OR "wearable devices" OR "wearable technology" OR "portable devices" OR "mHealth devices" OR "mobile health devices") |
| **1** | ("Chronic Pain"[mh] OR "persistent pain" OR "long-term pain" OR "recurrent pain") |

**Scopus**

| **3** | *#1 AND #2* |
| --- | --- |
| **2** | *TITLE-ABS-KEY("Wearable Electronic Devices" OR wearable OR "wearable devices" OR "wearable technology" OR "portable devices" OR "mHealth devices" OR "mobile health devices")* |
| **1** | TITLE-ABS-KEY("chronic pain" OR "persistent pain" OR "long-term pain" OR "recurrent pain") |

**IEEE Xplore**

| **3** | *#1 AND #2* |
| --- | --- |
| **2** | *("Wearable Electronic Devices" OR wearable OR "wearable devices" OR "wearable technology" OR "portable devices" OR "mHealth devices" OR "mobile health devices")* |
| **1** | ("chronic pain" OR "persistent pain" OR "long-term pain" OR "recurrent pain") |

**Engineering Village**

| **3** | *#1 AND #2* |
| --- | --- |
| **2** | *("Wearable Electronic Devices" OR wearable OR "wearable devices" OR "wearable technology" OR "portable devices" OR "mHealth devices" OR "mobile health devices")* |
| **1** | ("chronic pain" OR "persistent pain" OR "long-term pain" OR "recurrent pain") |
| **Web of Science** | |
| **3** | #1 AND #2 |
| **2** | TS=("Wearable Electronic Devices" OR wearable OR "wearable devices" OR "wearable technology" OR "portable devices" OR "mHealth devices" OR "mobile health devices") |
| **1** | TS=("chronic pain" OR "persistent pain" OR "long-term pain" OR "recurrent pain") |
| **Google Scholar** | |
| **1** | ("chronic pain" OR "persistent pain" OR "long-term pain" OR "recurrent pain") AND ("Wearable Electronic Devices" OR wearable OR "wearable devices" OR "wearable technology" OR "portable devices" OR "mHealth devices" OR "mobile health devices") |
